# Prosecutions for Mal-Practice in the State of New York

**Published:** 1847-08

**Authors:** Alden March

**Affiliations:** Albany


*Prosecutions for Mal-Practice in the State of New York.*

To the Editor of the Boston Medical and Surgical Journal.

Sir,—I saw, by a notice in a late No. of your Journal, that a physician in Delaware County, of this State, had been prosecuted for mal-practice in the treatment of a surgical case. Perhaps a brief history of the case, treatment and result, in connection with another of alleged mal-practice in surgery, disposed of last week, in the Washington County Circuit Court, may not be uninteresting, if not instructive to the medical profession.

In giving the report of these cases publicity in a widely-circulated medical journal, I trust the public interest may be subserved, and the junior members of the profession benefited. I desire not to wound the feelings of any professional man; and shall, therefore, avoid giving the names of the physicians charged with the mal-treatment of the following surgical cases.

Some time about the 11th of September, 1845, two young men, both "*posse men*," or in the employ of the State, during the late anti-rent difficulties, were wrestling; one of whom was about to be thrown upon the ground, and in the act of falling, thrust out his left hand, which met the ground rather outwards and backwards from his body, receiving not only the weight of his own body, but in all probability that of his antagonist; which resulted in a fracture of the external condyle of the humerus, and a backward dislocation of the ulna. In fifteen or twenty minutes after the receipt of the injury, the patient was placed in the hands of a young physician, who, I believe, regarded the accident a backward dislocation of the elbow.

By the testimony it appeared that he attempted to reduce it, by directing one person to make use of counter-extension from the humerus, midway between the shoulder and elbow, and another individual was directed to extend upon the fore-arm, by grasping the limb at the wrist; while the physician, by the aid of his own hands, endeavoured to crowd the articulating extremity of the humerus, situated in what should be the hollow of the arm, backwards. The extension and counter-extension was applied while the limb was in the *extended* or *straight* position; and after the application of considerable force, for some little time, there was an apparent yielding, when the physician would direct the limb to be slightly flexed, which twice resulted in something like a "chuck backwards." At last, on a third trial, extension and counter-extension was applied as in the other two instances, and the limb left nearly in the straight position, with little or no effort made to flex it. It was pronounced reduced; and the patient thought "it felt more like an arm." The degree of flexion can be pretty accurately estimated, when it is stated, that in order to make a sling sufficiently long to suspend the limb over the opposite shoulder, it required, or, at least, two handkerchiefs were tied together, and used in the ordinary way.

The physician saw the patient from time to time, for about thirty days; and up to near this period, said that "it was all right, or as near as it could be, except a little point of bone or process, which would come right by using the limb."

At the end of seven or eight weeks, the patient called on me at one of our Saturday cliniques at the Albany Medical College, with his elbow swollen, stiff, and in nearly the straight position. The lower end of the humerus could be felt quite distinctly at the fore part of the joint, and the olecranon projected unnaturally behind; in short, the ulna was, at that time, dislocated backwards. I made a vigorous, though unsuccessful, attempt to reduce it. In the great exertion employed to flex the limb, and the effort to dislodge the coronoid process from the olecranon fossa, the extreme point of the olecranon process gave way. The fore-arm, however, was brought to something more than a right angle with the arm, which would have been in a much more useful position, if, as we anticipated, a joint but little better than anchylosed, should be the result. By neglect on the part of the patient, and by the weight of the fore-arm, the limb at length returned to a state but little more flexed than at the time when I first saw it, and thus remained stiff and inflexible.

In something less than a year after the accident, the young man, by the advice of his friends (not medical,) commenced a suit for damages; charging, in his declaration, that the joint was then dislocated; that it had never been reduced, and consequently that he had, in a great degree, lost the use of his limb. That a backward dislocation of the ulna existed at the time of the trial, neither the defendant nor witnesses pretended to deny, and that a great share of the difficulty and loss of the use of the limb was to be attributed to the dislocation.

The counsellor for the plaintiff endeavoured to show, that the malpractice consisted in the fact, that the defendant did not make use of the most common and best means to reduce the dislocation; and that no evidence, at the time of the attempted reduction, was had, to show that the dislocated bone had been replaced.

One of the first questions put, on the part of the prosecution, was, "What is the present condition of the elbow-joint of the plaintiff; and what was the original nature of the injury?" All agreed, that there was a fracture of the external condyle, which presented its proper relation to the head of the radius, and that there was a dislocation of the ulna backwards. Some of the witnesses thought that the coronoid process of the ulna was broken off; and others, that the fracture, which carried away the external condyle, extended through the trochleal articulating surface of the humerus, so that by the action of the muscles, the coronoid process (provided that it was not broken off) was drawn, as it were, between the fragment and the continued shaft; or, in other words, that the articulating surface of the humerus was not sufficiently broad to sustain the ulna, after having been brought into its proper place. These two points, together with a feeble attempt to show that the patient had been imprudent in walking about too soon; in permitting a friend or nurse to dress it; and in riding on horseback, constituted the chief grounds of the defence.

On the part of the prosecution, after inquiring into the condition of the limb, nature of the accident, &c., the question was asked, "What is the evidence of reduction of a dislocated limb or joint?" All the witnesses, both for prosecution and defence, replied, "restoration of the form and shape of the joint, and freedom of motion.

The next question asked, was concerning the proper mode and means to be employed in reducing a backward dislocation of the ulna; and without a single exception, all replied, that it would be proper to apply extension and counter-extension while the elbow was in a *flexed* position. This principle was too *obvious* to be denied, or controverted, from the clear and conclusive demonstration made by the aid of the os humeri and ulna. By flexing the joint, the coronoid process was brought out, and forward from the deep olecranon fossa; and by extending it, it was made to lock into the deep olecranon fossa, and there retain its hold with great firmness.

The book authorities, with the exception of a single case given by Sir. A. Cooper, in his work on Dislocations, direct that the limb should be *flexed*, as by bending the joint around the knee, or around a bed post. It was agreed by all the medical witnesses, that if the limb was *flexed*, and, at the same time, if a *separating* force were applied to the extremities of the two bones involved in the dislocation, whether by the hands, or in any other way, such a dislocation as that under consideration might be reduced.

Another question asked the medical witnesses, was this: "If the coronoid process be broken off, would it not be proper, and the authorized practice, to place the limb in the extended position; or would it be correct practice for the treatment of the fracture, especially if there had been a backward dislocation of the ulna?" The question was also asked, "Should the limb be placed in the *semi-flexed* or *flexed position?*" To the former question all answered in the negative; and to the latter, the same reply was made by all the physicians, with the single exception of a young man who was then practising dentistry, and who testified that he was a graduate in medicine; had attended lectures in New York; and had there received instruction in the lectures of the Professor of Surgery to treat fracture of the coronoid process of the ulna, by placing the elbow-joint in the *semi-flexed position.*" I find no authority for this practice in any surgical work with which I am acquainted; nor have I learned that such is the doctrine taught in any other medical institution out of the city of New York. Perhaps the young surgeon might have been mistaken, or misunderstood the direction of his teacher. If so, I presume that the distinguished and able surgeon of New York, under whose instruction he claimed to have received such direction, will notify him of his mistake, and put him right upon this important principle.

Another question put on the side of the prosecution, was this. "Provided the coronoid process be broken off, and the ulna dislocated backwards; and provided, also, it had been reduced, in which position, whether in the *extended* or *flexed*, would the ulna be most likely to remain in its proper place?" Here, too, the universal answer was, "in the flexed position."

The above are the most essential and important facts as developed on the trial, by candid and fair witnesses; and the result was a verdict for the plaintiff of $450.

The prosecution was conducted in a most scientific and able manner by A. Tabor, Esq., of this city, assisted by the Hon. Mr. Wheeler, of Delaware County. Mitchell Sanford, Esq., of Greene County, conducted the defence, assisted by A. Parker, Esq., of Delhi. Mr. Sanford, who addressed the jury, displayed his usual tact, ingenuity and eloquence, in endeavouring to convince the minds of his hearers, that the young physician had neither done wrong, nor had neglected to do any thing that ought to have been done.

The Hon. Amasa J. Parker, of this city, the Circuit Judge, before whom the cause was tried, gave a brief, though clear and impartial charge, stating that the jury were to be governed by the law and the facts in the case before them. The principle of law, applicable to the case under consideration, was fully explained; and as the testimony to the facts in the case was so fresh upon the minds of the jury, he deemed it altogether unnecessary to repeat it.

In most of the prosecutions of physicians and surgeons for malpractice, it is fair to presume, from a pretty extended observation, that they originate in, or grow out of, an unwarrantable rivalry, or perhaps jealousy, between two neighbouring practitioners. In this case no such cause existed; on the contrary, it would seem as though all the medical profession, who testified on either side, were willing and anxious to protect the defendant, and the medical profession, so far as they could consistently with the solemn responsibilities of an oath resting upon them.

The report of the Washington County suit for mal-practice, may be expected next week. ALDEN MARCH.

*Albany, June, 1847.*

